# Body Composition in Chronic Liver Disease

**DOI:** 10.3390/ijms25020964

**Published:** 2024-01-12

**Authors:** Hiroki Nishikawa, Soo Ki Kim, Akira Asai

**Affiliations:** 1 Second Department of Internal Medicine, Osaka Medical and Pharmaceutical University, Takatsuki 569-8686, Osaka, Japan; 2 Department of Gastroenterology, Kobe Asahi Hospital, Kobe 653-8501, Hyogo, Japan

**Keywords:** body composition, liver, skeletal muscle, adipose tissue, sarcopenia

## Abstract

Body composition has recently been attracting people’s attention, not only from a cosmetic standpoint but also from the perspective of health and longevity. The body is classified into three components: fat, bone, and lean soft tissue, and it is common to see an increase in body fat and a decrease in total body muscle mass with aging. Aging-related loss of muscle mass and muscle function is referred to as primary sarcopenia, while sarcopenia caused by disease-specific conditions is referred to as secondary sarcopenia. On the other hand, the liver-muscle axis has been attracting attention in recent years, and it has become clear that the liver and the skeletal muscles interact with each other. In particular, patients with cirrhosis are prone to secondary sarcopenia due to protein-energy malnutrition, which is a characteristic pathophysiology of the disease, suggesting the importance of the organ–organ network. In this review, we would like to outline the latest findings in this field, with a focus on body composition in liver diseases such as liver cirrhosis, fatty liver disease, alcoholic liver disease, and hepatocellular carcinoma.

## 1. Introduction

The body is divided into three components: fat, bone, and lean soft tissue, and imbalances among these components can cause various diseases. The decline in muscle mass due to aging begins in the late 30s and 40s, and accelerates thereafter [1]. Aging-related loss of muscle mass and muscle function is referred to as primary sarcopenia, while sarcopenia caused by disease-specific conditions is referred to as secondary sarcopenia [1]. On the other hand, the liver–muscle axis has been attracting attention in recent years, and it has become clear that the liver and the skeletal muscles interact with each other [2]. Skeletal muscle is the largest store of glucose in the human body, and can be considered an endocrine organ, since it regulates glucose metabolism. Especially in cirrhotic patients, it is prone to secondary sarcopenia due to its characteristic pathophysiology and protein-energy malnutrition (PEM) [3]. The effects of alcohol on skeletal muscle, of fatty liver (FL) on skeletal muscle, of malignancy on skeletal muscle, and of gut–liver circulation on skeletal muscle are also recent topics of interest. The frequency of sarcopenia in liver disease varied markedly from report to report, ranging from 10 to 70% in Japan, partly due to the fact that the method of determination varied from report to report [3]. In addition, since sarcopenia is a strong prognostic factor in liver disease, the Japanese Society of Hepatology (JSH) developed sarcopenia assessment criteria specific to liver disease in 2016 [3]. This criterion has been cited many times in many countries, suggesting that the world is paying attention to this condition. (Note, however, that the reference values for determining sarcopenia are different between Asians and Westerners because of their different physiques [3,4].) With these backgrounds in mind, this review will outline the latest findings on body composition (especially skeletal muscle and fat) in various liver diseases. First, primary sarcopenia will be mentioned, followed by its association with body composition in patients with cirrhosis, FL, alcoholic liver disease, and liver cancer. Finally, the association between gut microbiota and body composition in liver disease will be noted. In this article, representative papers are cited as appropriate, using the key words “body composition” and “liver disease.”

## 2. Body Composition and Primary Sarcopenia

The body is composed of four major components: water, protein, fat, and minerals, and is classified into three elements: fat, bone, and lean soft tissue. These imbalances can lead to lifestyle-related diseases and chronic disease-related symptoms such as obesity, diabetes, hyperlipidemia, osteoporosis, edema, malnutrition, physical dysfunction, and falls [5]. Body composition, including body weight and body fat, has attracted people’s attention in recent years, not only from a cosmetic standpoint but also from the perspective of health and longevity [6]. Weight gain or loss depends on the balance between energy intake through diet and energy expenditure through metabolism and exercise.

Generally, aging changes in body composition include an increase in body fat and a decrease in total body muscle mass. In other words, elderly people have both “obesity” and “emaciation” problems at the same time. The decline in muscle mass due to aging is said to begin in the late 30s and 40s, followed by an accelerated decline [1]. This is a phenomenon that can occur for anyone, and is difficult to avoid [1]. Primary sarcopenia has been associated with decreased stimulation of the central nervous system, decreased physical activity, decreased sex and growth hormones (GHs), decreased protein intake, and increased inflammatory response [1]. In particular, inflammation has negative effects on skeletal muscle, either directly via proteolysis or indirectly via a reduction of GH and insulin-like growth factor (IGF)-1 via IL-6 and others [7]. A number of studies have shown that inflammatory markers are associated with physical decline in the elderly [7]. IL-6 and C reactive protein (CRP) are called aging-related “geriatric” cytokines, which increase with aging and are produced by trauma, stress, and infection, and have many physiological effects [8]. Muscle protein-related factors in primary sarcopenia are listed in Table 1.

There are also many reports of a selective decrease in type II fibers (fast-twitch muscle fibers) with aging [1,9]. Since type II fibers are associated with glycolytic capacity and insulin resistance (IR), type II fibers may also be the basis for the association between sarcopenia and diabetes development [1,9]. Most of the body’s glucose stores are in skeletal muscle, and a decrease in muscle mass with aging is thought to directly affect the function of glucose metabolism [10]. Furthermore, muscle is a tissue directly attached to bone, and it has long been noted that sarcopenia affects bone density and bone metabolism, forming the pathology of so-called “osteosarcopenia” [11]. In a study of 126 cirrhotic patients by Saeki et al., 24 (19%) had osteosarcopenia, and osteosarcopenia has also been shown to be a poor prognostic factor [12]. On the other hand, as mentioned earlier, a characteristic of aging-related changes in body composition is an increase in body fat mass in addition to a decrease in muscle mass, but such aging-related changes in body composition show large individual differences, and these changes may be observed simultaneously. In other words, despite a low body mass index (BMI) due to low muscle mass, body fat ratio and abdominal circumference may be rather high, and such a condition is called “sarcopenic obesity” [13]. Sarcopenic obesity is not only associated with physical dysfunction, but also with progressive metabolic disorders and atherosclerosis, which may increase the risk of developing cardiovascular disease [14]. Although the establishment of diagnostic criteria is necessary for the development of clinical research on sarcopenic obesity, there are still no established diagnostic criteria for sarcopenic obesity in the Japanese population.

## 3. Body Composition in Liver Cirrhosis

Recently, the liver–muscle axis has been the focus of attention, and it has become clear that the liver and the skeletal muscles interact with each other [2]. It has been shown that the rate of annual loss of muscle mass is approximately twice as high in cirrhotic patients as in the average elderly Japanese individual [15]. This is partly due to the fact that cirrhotic patients are more likely to have PEM. The early morning fasting burn (carbohydrate–fat–protein) ratio of the three macronutrients is about = 5–6:2–3:2 in healthy subjects, whereas, in advanced cirrhosis, it is about 1–2:6–7:2, with significant endogenous fat burning [16]. In other words, in advanced cirrhotic cases, the patient is in an energy state similar to starvation [16]. In cirrhosis, IR is increased and branched-chain amino acids (BCAAs) are used as an energy source, and BCAAs are decreased because they are used for ammonia processing in the skeletal muscle. Of the BCAAs, leucine is important for muscle protein synthesis [17].

In 2016, the JSH published criteria for secondary sarcopenia specific to liver disease [3], and several years later, a JSH working group conducted a revision process. A study of the prognosis of 1624 patients with liver disease showed that grip strength was a prognostic factor, and the reference value for grip strength in men was revised from 26 kg to 28 kg (women were not revised from 18 kg) [18]. These reference values of 28 kg for men and 18 kg for women are consistent with those proposed by the Asian Working Group for Sarcopenia (AWGS) [19]. In actual clinical practice, it is also important to reliably pick up sarcopenic patients, and there are simple screening methods such as the finger-circle test and SARC-F [20]. The SARC-F is a five-item questionnaire, with a score of four or higher on a ten-point scale suggesting a suspected sarcopenia diagnosis, and is recommended for use in screening for sarcopenia in the AWGS criteria [19], but it should be noted that there is a sensitivity problem (more cases may be missed) [21]. In our study, among 1282 patients with gastrointestinal diseases who underwent SARC-F questionnaires, the percentage of patients with SARC-F scores of four or higher was 17.5% for upper gastrointestinal diseases, 12.0% for lower gastrointestinal diseases, 17.3% for hepatobiliary and pancreatic diseases and 13.7% for liver diseases [22]. Considering that the frequency of sarcopenia in independent older adults is 7–10% [23], gastrointestinal diseases are at high risk for the development of sarcopenia secondary to the disease condition itself.

On the other hand, a meta-analysis of the prognostic impact of sarcopenia in cirrhotic patients was published in a journal [24]. It is significant that four papers from Japan (Hanai et al., Hiraoka et al., Nishikawa et al., Hamaguchi et al.) were cited in the meta-analysis. In this meta-analysis, the following points were emphasized: (1) the overall sarcopenia complication rate in cirrhotic patients was 37.5%, (2) the frequency of sarcopenia increased with worsening Child-Pugh score, (3) sarcopenia was more common (49.6%) in patients with alcoholic cirrhosis, and (4) the 5-year survival rate is 45.3% for patients with sarcopenia and 74.2% for those without sarcopenia. (5) In forest plots, the lower limit of the 95% confidence interval for the hazard ratio (HR) of sarcopenia on mortality exceeds one in all articles, indicating that sarcopenia is a definite prognostic factor in cirrhotic patients [24].

In the flowchart for nutritional therapy in the Japanese guidelines for the treatment of cirrhosis 2020, patients are initially screened for serum albumin levels, Child-Pugh classifications, and the presence of sarcopenia (using JSH criteria [3]), and even if none of these are met, in patients with a low BMI (<18.5 kg/m^2^), nutritional dietary guidance and general enteral nutrition are recommended, taking into account the risk of sarcopenia. If any of these conditions are met, the administration of BCAA granules or enteral nutrition for hepatic failure is recommended, taking into account serum albumin levels and complications such as ascites and encephalopathy, in addition to the evaluation of food intake and nutritional status [25]. International guidelines also state that cirrhotic cases with low BMI are at high risk of sarcopenia, and recommend detailed pathological evaluation and nutritional intervention [26]. As for separate meals, they are also strongly recommended for improving one’s non-protein respiratory quotient. It has been reported that the administration of BCAA granules to cirrhotic patients significantly improves homeostasis model assessments for insulin resistance (HOMA-R), and significantly suppresses the cumulative carcinogenesis rate in cirrhotic patients with BMI > 25 kg/m^2^, who often have IR [27,28].

## 4. Boby Composition in Fatty Liver

The number of FL patients in Japan is increasing, with approximately 25 million people currently suffering from FL, and approximately 20% of non-alcoholic fatty liver disease (NAFLD) patients are said to have FL without obesity (i.e., lean NAFLD) [29]. Adipose tissue produces inflammatory proteins and cytokines such as CRP, TNF-α, IL-6, and IL1β, forming a chronic inflammatory environment that leads to muscle atrophy and sarcopenia [30]. Adipose tissue also produces numerous hormones and bioactive substances such as adiponectin. Adiponectin is present in large amounts in the blood, decreases with fat accumulation, and increases with weight loss [9]. Adiponectin improves systemic insulin sensitivity and stimulates free fatty acid oxidation and glucose uptake in skeletal muscle and adipocytes via AMP-activated protein kinase [31]. Adiponectin receptor-1 and adiponectin receptor-2, receptors for adiponectin, are present in the skeletal muscle, and adiponectin decreases with aging [31].

Overseas studies have reported that NAFLD is associated with a higher rate of sarcopenia than other chronic liver diseases such as hepatitis C virus and hepatitis B virus, with 40% of NAFLD [32]. Skeletal muscle loss and increased body fat mass have been reported to be risk factors for NAFLD development and NAFLD exacerbation [33,34]. Conversely, NAFLD is a risk factor for the development of sarcopenia, and therefore NAFLD and sarcopenia have a bidirectional relationship [35]. Individuals with NAFLD and without obesity are also prone to sarcopenia, and should be treated with caution [36]. The balance between fat and skeletal muscle is also very important: in our study of 1186 men and 1441 women (health checkup subjects), fat mass to fat-free mass ratio was strongly correlated with HOMA-R, and a multivariate analysis results showed that fat mass and fat-free mass were not independent factors related to HOMA-R, but fat mass to fat-free mass ratio was shown to be an independent factor [37]. In the management of diabetic patients, it is important to have an awareness of the fat–skeletal muscle linkage [37].

Although NAFLD has served as an anchor point for clinical practice and trials, substantial concerns have been raised about its use due to the inherent drawbacks of being exclusionary and stigmatizing, prompting a search for new nomenclature. In April 2020, distinguished hepatologists from all over the world met and discussed the new disease concept of metabolic dysfunction-associated fatty liver disease (MAFLD) [38], which is now becoming more common in the medical community [39]. The diagnostic criteria for MAFLD are FL and one or more of the following three conditions (with or without a history of alcohol or other causes of liver disease): (1) overweight and obesity (BMI ≥ 23 kg/m^2^ in Japanese); (2) type 2 diabetes; and (3) thin/normal weight with two or more metabolic abnormalities (hypertension, visceral fat accumulation, glucose intolerance, dyslipidemia) [38]. MAFLD is a new disease concept that combines FL and metabolic disorders, and was proposed to detect and treat “dangerous FL” at an early stage [38]. MAFLD is also a disease definition that is designed to be prognostic for FL patients, not only for the liver, but also for the body as a whole. MAFLD is even more likely to progress in the presence of metabolic abnormalities such as obesity and type 2 diabetes [40]. The disease concept of MAFLD is expected to help address FL disease from multiple perspectives, including gastrointestinal, cardiovascular, and endocrine metabolism [41]. The latest nomenclature proposed steatotic liver disease (SLD) as an umbrella term, consisting of several subcategories based on the presence of five cardiometabolic risk factors, in addition to other etiologies such as alcohol intake. Patients with SLD who have cardiometabolic risk factors are further classified as metabolic dysfunction-associated SLD (MASLD) [42].

A Korean report on MAFLD and muscle mass showed that MAFLD and a loss of skeletal muscle mass were closely correlated, with diabetes-associated MAFLD being associated with the greatest loss of skeletal muscle mass, and patients with advanced liver fibrosis had a significantly higher rate of loss of skeletal muscle mass than those without the advanced disease [43]. Combining appendicular skeletal muscle mass and diabetes can also predict the degree of hepatic steatosis in MAFLD [44]. IR and body fat mass are significantly interrelated in MAFLD patients [45]. A close correlation between MAFLD and low skeletal muscle mass has also been reported in patients with hepatitis B [46].

In our study of MAFLD and skeletal muscle mass (median age and BMI were 55 years and 25.4 kg/m^2^ in men (2014 cases), and 57 years and 25.4 kg/m^2^ in women (949 cases), respectively), fat-free mass index was strongly correlated with muscle mass index in both men (correlation coefficient *r* = 0.999) and women (*r* = 0.999). The proportion of patients with a fat-free mass index <18 kg/m^2^ in men and a fat-free mass index <15 kg/m^2^ in women (the threshold for skeletal muscle loss) was significantly stratified by age, BMI, severity of FL, and FIB4 index. In men, BMI (*p* < 0.0001), fat mass index (*p* < 0.0001), and waist circumference (*p* = 0.0050) were found to be significant factors associated with fat-free mass index in the multivariate analysis. In women, BMI (*p* < 0.0001) and fat mass index (*p* < 0.0001) were found to be significant in the multivariate analysis, indicating a strong correlation between body fat mass and muscle mass in MAFLD patients [47].

## 5. Body Composition and Alcohol Intake

**Figure 1:**
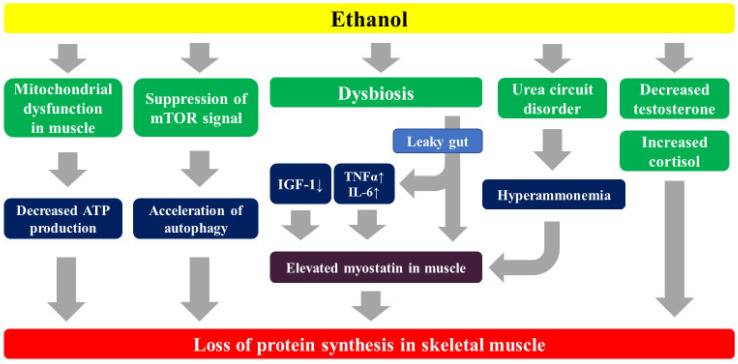
The effect of ethanol on the skeletal muscle.

Chronic overconsumption of alcohol causes fatty degeneration in the liver first, followed by a gradual progression of hepatic fibrosis. Ethanol acts directly and indirectly on muscle cells, causing skeletal muscle atrophy and a loss of function [48]. There are six main possible mechanisms: (1) ethanol inhibits mitochondrial function and ATP production in skeletal muscle cells [49]; (2) ethanol and its metabolite acetaldehyde suppress mammalian target of rapamycin (mTOR) signaling, inhibit muscle protein synthesis, and promote autophagy [50]; (3) ethanol affects ZO-1 and claudin-1 binding, two important proteins in intestinal epithelial tight junctions [51], and also promotes the in vivo influx of endotoxins, and induces the production of inflammatory cytokines such as TNF-α, leading to increased myostatin in skeletal muscle and inhibiting the synthesis of muscle proteins (described later) [52]; (4) ethanol reduces gonadal function and decreases the production of testosterone, an anabolic hormone that activates muscle satellite cells and promotes muscle protein synthesis [53]; (5) alcohol intake increases cortisol, which is also a stress hormone, and cortisol breaks down skeletal muscle [53]; and (6) acetaldehyde inhibits ornithine transcarbamylase, a known rate-limiting enzyme in the urea circuit, resulting in increased blood ammonia levels [52] (Figure 1). Alcohol abstinence and sobriety are considered effective means of improving sarcopenia. Alcohol consumption and muscle mass show a strong inverse relationship regardless of gender [54].

According to the annual trends in the causes of cirrhosis reported by the JSH, until 2007, hepatitis C accounted for 58.6%, hepatitis B for 13.6%, alcohol for 13.7%, and non-alcoholic steatohepatitis (NASH) for 2.0%, while, after 2014, hepatitis C, hepatitis B, alcohol, and NASH accounted for 40.2%, 9.0%, 24.9%, and 9.1%, respectively, and the share of alcoholic cirrhosis increased by more than 10% [55]. The latest data presented at the 59th Annual Meeting of the JSH in 2023 showed that alcohol accounted for 28.8%, hepatitis C for 27.1%, and NASH for 12.7% from 2018 to 2021, indicating that a significant change in the causes of the disease had occurred, albeit at a preliminary stage [56]. While there have been significant advances in antiviral therapies such as direct-acting antivirals and nucleoside analogues, the difficulties in managing alcoholic cirrhotic patients who have difficulty abstaining from alcohol or moderating their drinking, and the increase in FL disease, which is estimated to account for over 20% of the Japanese population, may have been highlighted. These trends may become increasingly evident in the future.

Saeki et al. reported that of 181 cirrhotic cases, 64 (35.4%) were alcoholic and younger than non-alcoholic ones (median: 61.5 vs. 72 years, *p* < 0.001). The frequency of sarcopenia was 18.8% vs. 32.5% (*p* = 0.048) for alcoholic and non-alcoholic patients, and the frequency of sarcopenia increased with increasing age [57]. On the other hand, as noted earlier, in a meta-analysis on cirrhosis and sarcopenia reported from overseas, sarcopenia was complicated in 49.6% of alcoholic cirrhosis, while the sarcopenia complication rate was 33.4% in non-alcoholic cirrhosis, which was considerably lower [24]. These results differ from those reported earlier by Saeki et al., and suggest that there are racial differences in alcohol-degrading enzymes, and differences in the amount of alcohol consumed.

Myostatin is a myokine that inhibits muscle protein synthesis. Excessive alcohol consumption adversely affects skeletal muscle protein metabolism and increases myostatin expression in muscle [58]. In alcoholic cirrhotic patients, the incidence of hepatocellular carcinoma (HCC) was shown to be significantly higher in patients with higher serum myostatin levels compared to those with lower serum myostatin levels (hazard ratio (HR) = 7.53) [59]. We were the first in the world to report that a higher serum myostatin level is an adverse prognostic factor in cirrhotic cases [60]. Serum myostatin levels also positively correlate with serum ammonia levels [60]. Serum zinc levels are often low in cirrhosis, and serum zinc levels and myostatin levels show an inverse correlation [60]. In patients with advanced alcoholic cirrhosis, hyperammonemia due to decreased ammonia clearance may lead to high levels of myostatin in muscle, resulting in the suppression of skeletal muscle protein synthesis and sarcopenia. Sato et al. reported that skeletal muscle mass and endotoxin levels were negatively correlated in patients with alcoholic cirrhosis (*r* = −0.67, *p* < 0.0001), and that hyper-endotoxemia due to alcohol-induced tight junction disruption is strongly associated with the development of sarcopenia in alcoholic cirrhosis [61].

## 6. Body Composition, Hepatocellular Carcinoma, and Cancer Cachexia

The condition called cancer cachexia has been known since the time of Hippocrates, who taught that food is the essence of medicine, and the word cachexia is derived from the Greek words kakos and hexis (bad condition). Cancer cachexia is strongly associated with symptoms that reduce patients’ quality of life, such as weight loss, anorexia, and fatigue, and is estimated to occur in 50–80% of advanced cancer patients and to account for 20% of cancer-related deaths [62]. In an observational study using Fearon et al.’s diagnostic criteria for cancer cachexia, the prevalence of cachexia was the highest in pancreatic cancer (88.9%), and totaled 50% in patients with HCC [63]. Proteolysis-inducing factor (PIF) secreted by cancer cells inhibits protein synthesis and accelerates proteolysis in skeletal muscle [64,65]. Lipid-mobilizing factor (LMF) secreted by cancer cells promotes lipolysis [65]. Inflammatory cytokines such as TNFα (1) accelerate proteolysis via the ubiquitin proteasome pathway (UPP), and (2) enhance glycogenesis in the liver via IR [66,67]. An increased consumption of glucose by cancer cells depletes liver glycogen, further increasing glycogenesis and accelerating fat breakdown and skeletal muscle degradation [66,67].

Recent meta-analyses have shown that sarcopenia is an adverse prognostic factor for HCC [68]. Guo Y et al. reported that 41.7% of HCC patients had sarcopenia, with a HR of sarcopenia for survival = 1.93, a HR for tumor recurrence = 1.75, a HR for response rate = 0.37, and a HR for adverse events = 2.23, all indicating a strong impact of sarcopenia in patients with HCC [68]. HCC is associated with cirrhosis at a high rate, but it should be noted that cirrhosis can easily be complicated by cachexia, whether in the compensated stage or with early stage HCC. Rich et al. found pre-cachexia in 201 (33.3%) of 604 HCC cases and cachexia in 143 (23.7%). In their results, by the HCC stage, 19.0% of BCLC stage 0/A, 23.5% of BCLC stage B, 34.7% of BCLC stage C, and 34.0% of BCLC stage D had cachexia. In addition, 41.8% of Child-Pugh A, 46.1% of Child-Pugh B, and 12.1% of Child-Pugh C patients had cachexia, and cachexia was reported to be an independent poor prognostic factor in patients with HCC [69].

## 7. Body Composition and Gut Microbiota in Liver Disease

There are approximately 1000 types of bacteria, or 100 trillion bacteria, known to live in the intestines, and they are composed of three major groups: good bacteria, bad bacteria, and bacteria in between that are neither. These bacteria are closely related to each other and are intricately balanced. Recent advances in next-generation sequencer and metabolomic analysis technologies have revealed that factors derived from the intestinal tract (e.g., intestinal bacteria) play a very important role in the development and aggravation of liver diseases via the enterohepatic circulation [70].

Excessive alcohol consumption (described earlier) and high-fat diets disrupt the intestinal barrier and strongly affect not only the composition of the gut microbiota, but also the microbe-associated molecular patterns (MAMPs) of bacterial metabolites and host interactions. As a result, it promotes the development of hepatitis, liver fibrosis, cirrhosis, HCC, and sarcopenia [71]. NASH is a pathological condition in which chronic inflammation is induced in a simple FL, leading to fibrosis and carcinogenesis, and the multiple parallel hits hypothesis is widely known as the mechanism of NASH [72]. Factors that cause inflammation include oxidative stress, inflammatory cytokines, lipotoxicity, IR, genetic predisposition and endogenous endotoxins, and their exposure to the liver plays an important role. In NASH, the intestinal defense mechanism is disrupted (i.e., leaky gut), and the increased influx of lipopolysaccharide (LPS) derived from intestinal bacteria into the liver results in the excessive production of inflammatory cytokines such as TNF-α via TLR4 signaling in Kupffer cells, which promotes liver fibrosis in NASH [73]. Exogenous endotoxin load also promotes liver fibrosis [74]. Dysbiosis in the gut promotes the induction of myostatin in skeletal muscle via LPS activation, and is a risk for the development of sarcopenia [75]. It is interesting to note that in the feces of cirrhotic patients, there is an increase in oral commensal bacteria, as well as an increase in Proteobacteria (Gram-negative bacteria with LPS) [76]. Hyperammonemia associated with decreased ammonia clearance due to cirrhosis also promotes the induction of myostatin in skeletal muscle, and is a risk factor for the development of sarcopenia [60]. Rifaximin enhances muscle protein synthesis capacity in cirrhotic rats via its ammonia-lowering effect in blood and muscle [77]. Constipation is a risk factor for hepatic encephalopathy (HE), and constipation and dysbiosis are closely correlated [78]. Trace element deficiencies, such as hypozincemia associated with cirrhosis, may also cause a disruption of intestinal defense mechanisms, leading to the development of sarcopenia [79,80,81]. HE improves when cirrhotic patients with HE are treated with fecal microbiota transplantation from healthy individuals [82]. Decreased grip strength correlates closely with covert HE, and decreased grip strength is a risk factor for the development of overt HE [83].

## 8. Closing Remarks

This review focuses on the relationship between liver disease and body composition. Since the liver is a central organ in metabolism, it is considered to be closely related to body composition. The strength of our article is that it presents the latest findings across a wide range of liver diseases. Conversely, however, this does not allow for a detailed reference to the relationship between individual liver diseases and body composition. Finally, we would like to emphasize the importance of approaching liver diseases with body composition in mind.

## Figures and Tables

**Table 1:** Primary sarcopenia and muscle protein-related factors.

Increase in muscle protein synthesis
1. Increased protein intake
2. Increased physical activity
3. Increased sex and growth hormones
4. Increased insulin-like growth factor 1
Decrease in muscle protein synthesis
1. Aging
2. Decreased protein intake
3. Decreased physical activity
4. Decreased sex and growth hormones
5. Increased inflammatory cytokines such as IL-6 and TNF-α
6. Decreased stimulation of the central nervous system

## Abbreviations

PEM: protein-energy malnutrition, FL; fatty liver, GH; growth hormone, IR; insulin resistance, IGF-1; insulin-like grow factor, CRP; C reactive protein, BMI; body mass index, JSH; Japanese Society of Hepatology, AWGS; Asian Working Group for Sarcopenia, HR; hazard ratio, HMA-R; homeostasis model assessment for insulin resistance, NAFLD; non-alcoholic fatty liver disease, MAFLD; metabolic dysfunction-associated fatty liver disease, SLD; steatotic liver disease, NASH; non-alcoholic steatohepatitis, HCC; hepatocellular carcinoma, LPS; lipopolysaccharide, HE; hepatic encephalopathy.
